# Key elements of networking in life sciences: collective creative thinking and team work

**DOI:** 10.3325/cmj.2015.56.75

**Published:** 2015-04

**Authors:** Sandor G. Vari

**Affiliations:** International Research and Innovation Management Program Cedars-Sinai Medical Center, Los Angeles, CA, USA & RECOOP HST Association

In 2006, Cedars-Sinai Medical Center (CSMC) with eleven Central and Eastern Europe (CEE) universities and academic organizations from six countries (Croatia, Czech Republic, Hungary, Romania, Slovakia, and Ukraine) formed the Regional Cooperation for Health, Science and Technology Consortium. In 2012, CSMC converted the Consortium into the Association for Regional Cooperation in the Fields of Health, Science and Technology (RECOOP HST). Members of the Association are from nine countries (Croatia, Czech Republic, Denmark, Hungary, Poland, Romania, Slovak Republic, Ukraine, and USA). The challenge of the construction of the Association was the integration of individual research goals into team research strategies. Building collaborative research work demands comprehensive knowledge of the economic and cultural environment, even the actual political climate, and foremost the funding mechanism that the participating organizations are challenging and utilizing. Also it is very important to have resources for face-to-face and networking meetings, annual scientific review conferences, and project management. Cedars-Sinai Medical Center provided such a support for RECOOP HST Association and that was the key to success (1).

Building a large-scale research collaboration takes time and it does not depend only on financial support but more on common research interests, well established communication among the scientists, open discussions initiated by the team leaders, and on research managers who have experience in laboratory and clinical research. The network managers must have comprehensive but not necessarily deep knowledge of the research fields where the team works and collaborations are taking place. They should have excellent managerial and good communication skills to assist the participating researchers in the realization of their research goals in network research. The bottom line is that the manager can convince the researchers in the network to mobilize the individual partner’s knowledge and resources for the team research they agreed on. The coordination of the research plan accepted by the network needs easy-to-manage logistics from animal housing, experimental set up, sample and laboratory supply transfer, and exchange of young scientists, PhD students, and post docs (2).

Collectively funding research was started in the 20th century by National Agencies (National Institutes of Health) and International Organizations (European Union via European Commission). The research funding in the 21th century depends on the national and global economic and research institutions, and private research foundations. One of them is the Organisation for Economic Co-operation and Development (OECD) assembling the world's 34 largest market-oriented economies. OECD continuously analyzes the research funding opportunities and has recently reported that the resources for research are not growing with the same dynamic in the US and in the EU as in the BRIICS (Brazil, Russia, India, Indonesia, China, and South Africa), and overall worldwide trend is lower than predicted ten years ago. The annual growth in research and development (R&D) spending across the 34 OECD countries in the four-year period of 2008-12 was 1.6%, which is half the rate of 2001-08.

The United States has long been the frontrunner and still is at the forefront of cutting-edge science, technology, and innovation. OECD report noted that the US lead is narrowing regardless of its topnotch universities and global technology corporations. In the meantime China's R&D spending from 2008 to 2012 doubled. European countries are diverging in R&D since some of them moved closer to their R&D/GDP targets (Denmark and Germany), while the CEE Countries, Greece, Portugal, and Spain fall further behind. The other important trend is that in most European countries only 10% to 20% of business R&D is funded with public money, using diverse investment instruments. Therefore it is predictable that higher number of researchers will turn to EU funds to maintain their competitiveness (3).

In the US from the beginning of public research funding, a bottom up model was established, funded by government agencies and favoring researcher-originated projects over thematically defined grants to promote technological and scientific innovation. In 2005 *Nature* investigated the funding process in the USA and concluded that those who influenced the funding process at one of the agencies, the National Institutes of Health, are not among those who publish the most valuable scientific literature (4,5).

In the European Union, the common practice is the top down model. This model, based on political decisions by the European Parliament, defines the priorities, which are executed by the European Commission (EC) via research programs called Framework Programmes and the latest HORIZON2020. Through successive enlargement, the Union has grown from the six founding states or the “inner six” to the current 28 members (6). In the selection of the funded research projects, EC tries to keep some geopolitical balance with requirements like the creation of research consortium for multinational teams. The major problem with this type of networking is that it is not created based on the demand of the participating researchers to share knowledge and resources to achieve their research goals. On the contrary, it is forced by the contract signed by the consortium that was created to receive the research grant.

Despite of this Pan-European approach, the CEE disadvantage is still strong. Researchers from CEE have little chance of getting a European Research Council (ERC) grant, since ERC funds mainly go to the 15 EU member states (EU 15) that became members before the 2004 enlargement. In 2013 about three out of four ERC starting grants for young scientists (222 out of 300) went to researchers hosted by institutions in the UK, Germany, France, and the Netherlands (7). We are witnessing the same in life sciences and health research. This is because in health research, according to the EC’s own report, most of the funds currently go to EU 15. The EU 15 received 34 times more health research funding under the FP7 than the newest 13 member states. This difference could not be accounted for by the differences in population size, GDP, or contributions to the budget. In fact, those 13 member states received less money from the FP7 than the rest of the world received from EU (8).

Funding is necessary but the most important element of the research networking is based on creative thinking. Moreover, we have to overcome the reality that most scientists are self-directed and that they desire to make discoveries independently. Creating trust within the network and making the participants believe that the success will be theirs is very important. Personal meetings can build trust and make communication easier and smoother. Therefore, it is an imperative to develop best practices for managing of intellect in order to build and sustain a competitive teamwork. In the research network building, the most vital tasks are to change the dynamic of the participating organizations, boost creativity, and enable research organizations to inspire and engage their scientists in team work. In this way, it is possible to integrate individual disciplinary models into multidisciplinary groups. Network managers are key drivers in the process of the collaborative works. Managers have to encourage teams to use interdependent efforts in moving the project from the developmental stage to the successful research projects (9).

Systematic understanding of how to consistently generate successful inter- and transdisciplinary research teams is a difficult task. The central element of the collaborative research is the development of shared vision. The engagement with laboratories, and bringing those laboratories into a network can be done with transformative learning of teamwork and by understanding the priorities of their scientific field. The integration and synthesis of knowledge and research knowhow of the participating organizations across disciplines can lead to new conceptualizations of research goals and multidisciplinary science in research networks. This is a challenging issue since the members of the research network have to leave the comfort zone of “chamber science,” but on the other hand it will enable their innovative thinking, which leads to a new vision of research, and can only be achieved through collaborations. For cross-disciplinary collaboration the individual research plans must be tuned and restructured to mesh with other individual research plans. The modifications should be discussed extensively during project meetings and slowly the collective thinking may emerge. It is very important to ensure that everyone knows how their contribution fits into the team vision, and understands the tasks being carried out in the network project. Also, the participating scientists have to understand that each member depends on the group, and building trust within the group is the key to success. At the same time, it is necessary to empower team members to be creative and autonomous (10).

Strategy implementation is the most valuable managerial skills, which requires steady and persistent work with the individual scientists and laboratories. The successful strategy establishes trust, provides support to sustain members’ competitive advantages, demonstrates that they could optimize their core competencies with the collaborative work, and strengthens their organizational capability. Scientists at all levels must be involved in the implementation process of the research network’s strategies. During this evolution of research networks, managers and network leaders must motivate other researchers to become leaders in their respective specialized research area to ensure that the networks will have continuity with the new generation of network leaders and managers (11).

## References

[1] Melmed S, Vari SG (2014). Challenges in life sciences and health systems in the 21st century.. Croat Med J.

[2] Puljak L, Vari SG (2014). Significance of research networking for enhancing collaboration and research productivity.. Croat Med J.

[3] Science OECD. Technology and Industry Outlook 2014 reviews. Available from: http://www.oecd.org/sti/oecd-science-technology-and-industry-outlook-19991428.htm. Accessed: April 8, 2014.

[4] (2012). Editorial. Moving bottom-up science closer to the top. Nature Methods.

[5] Nicholson JM, Ioannidis JPA (2012). Research grants: Conform and be funded.. Nature.

[6] Archick K. The European Union: questions and answers. Congressional Research Service. January 13, 2015.

[7] The Guardian Higher Education Network. Anonymous academic. European research funding: it’s like Robin Hood in reverse. November 7, 2014 Friday 02.00 EST. Available from: http://www.theguardian.com/higher-education-network/2014/nov/07/european-research-funding-horizon-2020*.* Accessed: April 8, 2014.

[8] Tatalovic ME. U’s Horizon 2020 should pay researchers in Eastern Europe the same salaries as in Western Europe. Business, Regional collaboration, Science funding, Science policy, April 19, 2013. Available from: http://www.scilogs.com/balkan_science_beat/eus-horizon-2020-should-pay-researchers-in*.* Accessed: April 8, 2014.

[9] Pennington DD, Simpson GL, McConnell MS, Fair JM, Baker RJ (2013). Transdisciplinary research, transformative learning, and transformative science.. Bioscience.

[10] Pennington DD. Cross-disciplinary collaboration and learning. Ecology and Society. 2008;13: 8. Available from: http://www.ecologyandsociety.org/vol13/iss2/art8/*.* Accessed: April 8, 2014.

[11] Boyce SY. Using intellectual capital and organizational capability to enhance strategic implementation for pharmaceutical firms. Journal of Business and Public Affairs. 2007;1:1. Available from: http://www.scientificjournals.org/journals2007/articles/1072.htm. Accessed: April 8, 2014.

